# Northwestern Branch of the Philadelphia County Medical Society

**Published:** 1904-04

**Authors:** 


### NORTHWESTERN BRANCH OF THE PHILADELPHIA COUNTY MEDICAL SOCIETY.

Meeting of March 10, 1904, Dr. Samuel Wolfe, President.

### A GENITO-URINARY SYMPOSIUM.

Papers Read: The Local Treatment of Gonorrheic Infections, by Dr. H. R. Loux, Chief of Genito-Urinary Clinic, Jefferson Medical College Hospital, Assistant Surgeon to Philadelphia Hospital; The Radical Cure of Senile Hypertrophy of the Prostate, based upon a study of 145 operations performed by the author, by Dr. Orville Horwitz, Professor of Genito-Urinary Surgery, Jefferson Medical College.

Discussion by Dr. Edward Martin, Professor of Clinical Surgery, University of Pennsylvania; Dr. H. M. Christian, Professor of Genito-Urinary Surgery, Medico-Chirurgical College; Dr. R. O. Kevin, Attending Surgeon Genito-Urinary Dispensary, Jefferson Hospital.

*Dr. H. R. Loux* read a paper entitled "The Local Treatment of Gonorrheic Infections."

Dr. Loux stated that, although a continuous service of eleven years in one of the largest genito-urinary clinics in America had afforded him unusual opportunities for observation, he had never written a paper upon the treatment of gonorrhea, because no method heretofore suggested proved, upon prolonged trial, to be an advance worthy of commendation. Clinical observation in thousands of cases convinced him that gonorrhea is too often grossly mistreated. He deprecated the use of strong, irritating injections because they aggravate the disease and damage the urethra, and stated that treatment of acute anterior urethritis by irrigation is to be condemned because it causes an extension of the disease by continuity; he quoted statistics, reasons and authoritative statements to show that these opinions represented the beliefs of the leading and most conservative genito-urinary surgeons.

The speaker stated that during the past year and a half the results at his clinic at the Jefferson Hospital and in private practice, had been much better than ever before; this statement he based upon the observation of several thousand cases of gonorrhea at all stages. The reasons for this improvement he ascribed to careful local treatment in which he abandoned, absolutely, the use of any drug as an injection which can cause the slightest irritation. Dr. Loux stated that, in a general way, his methods of treatment were as follows: For acute gonorrhea, he prescribes light diet, with very little meats, no fats, fruits or alcoholic beverages, but allows as much skimmed milk as the patient can drink. If the affection is confined to the anterior urethra, he prescribed the injection of two drachms of a ten per cent. solution of argyrol, held in the urethra ten minutes; this injection is made in the morning, at noon and at night. Internally, he prescribes capsules of copaiba, cubebs and sandalwood three times daily. This treatment is practiced for one week, during which time the discharge will almost if not entirely cease, there will be no pain or irritation by the injection or upon urination, and the gonococci will disappear.

If, at the end of one week, the urine remains continuously shreddy, a weak solution of astringents is employed, and of these drugs he preferred zinc sulphate, iodide, chloride, hydrastin or berberine muriate, but emphasized that these astringents should not be used during the first week of the disease and never in solutions sufficiently strong to produce pain or irritation.

If the two-glass test shows cloudy first and second portions of the urine, showing the presence of antero-posterior urethritis, he irrigates the anterior urethra with a warm solution of boracic acid in order to remove the accumulated secretions. Then he makes deep instillations of twenty per cent. argyrol solutions once daily or alternate days; the inflammation of the anterior urethra is treated in the manner already described.

The writer quoted from statistics of four hundred cases, treated since July, 1902, by the methods described, and stated the advantages as follows: simplicity; the relief afforded the patient from pain and irritation: the extreme rarity of complications; shortened duration of diseases, in that an average time required for cure in acute cases was twenty-one days, whereas by the older methods practiced at the clinic and in private work, the best average obtainable was forty-two days.

Dr. Loux then discussed the treatment of gonorrhea in the female, the methods of which are best described by an illustrative case: A. G., aged twenty-one, came under observation June 5, with acute vaginitis, endometritis and urethritis, for which she had been under treatment in New York for one week. Typical symptoms of gonorrhea were present; microscopical examination positive. The vagina was dilated to full extent by means of speculum. To every portion of the vaginal mucous membrane a 50 per cent. argyrol solution was applied, and the same to the urethra by means of a cotton-tipped probe. The interior of the uterus was then freed of accumulated secretions by means of a cotton-wrapped applicator, after which the 50 per cent. argyrol solution was applied to the cervix and the body of the uterus. These applications are repeated two or three times so as to fill the uterine cavity with the solution. This local treatment was carried out every second or third day. After eight days no gonococci could be found. For home treatment the patient was ordered vaginal douches of from two to four quarts of hot boracic acid or normal salt solutions taken in the recumbent posture. On June 20th there was no discharge or other symptoms of gonorrhea and the patient was discharged. On

June 29th she was attacked with acute appendicitis, for which he operated and removed a sloughing appendix. For the subsequent five weeks, during which she was in bed in the hospital recovering from the operation, he made observation every few days of her genito-urinary organs and there was no symptom of gonorrhea. The patient made an uninterrupted recovery and left the hospital.

In discussing *chronic conditions* which result from a neglected or improperly treated gonorrhea, Dr. Loux stated that care should be exercised in adapting methods and means to avoid irritating the already-damaged urethral structures. He emphasized the necessity of making routine use of the endoscope and determine its nature.

Chronic follicular urethritis is readily recognized by endoscopic examination and by palpation of the enlarged follicles over a bougie, and is treated by gradual dilatation of the urethra by means of bougies, massage of the enlarged follicles, and by the local application of twenty-five to fifty per cent. argyrol solution to the individual enlarged follicles as revealed by the endoscope. This treatment is carried out three or four times a week and is by far the most satisfactory method he had ever found.

Most cases of chronic gleet are due to ulcerative conditions of the urethra, and in the management of these the endoscope is indispensable. After determining the exact location of the individual ulcerations the method of treatment depends upon whether the ulcerations are sharply localized or whether there is a co-existent general hyperæmia of the urethra. In the former case, applications of fifty per cent. argyrol solution (through the endoscopic tube) to the ulcerations should be made at least three times a week. If general hyperæmia exists, the use of mild astringents should precede the topical application of argyrol, in order to rid the urethra of the muco-purulent accumulations. From four to six weeks of this treatment, with care in the use of instruments, will heal the ulcerations and cure the gleet in the large majority of cases.

Another very common condition is the reduction in the lumen of the urethra by inflammatory exudate, occasioned by repeated attacks of gonorrhea or a primary case of long duration. In these cases, endoscopic examination shows the seat of beginning stricture and the presence of more or less localized inflammation. The management of these cases is extremely important because of the certainty of the occurrence of organic stricture unless the patient agrees to a several weeks' course of treatment. He should report every third or fourth day for the passage of bougies of gradually increasing sizes, followed, if active inflammation exists, by the deep instillation or topical application of twenty-five per cent. argyrol solution, depending upon whether the inflammation is circumscribed or more or less diffuse.

Dr. Loux summarized his paper as follows: Concerning acute gonorrhea: 1. He would strongly deprecate the treatment of acute anterior urethritis by means of irrigation, because of the danger of spreading the disease to the posterior urethra. 2. Irritating injections of any kind should never be used in acute gonorrhea because of the certainty of occurrence of a mixed infection and the extension of the disease by contiguity to the urethral follicles. 3. Argyrol, as a non-irritating gonococcide, with a specific effect in allaying the symptoms of inflammation, is the drug of choice for injection, and may be used in any strength and at any stage of the disease. 4. Astringents, such as zinc, hydrastin, bismuth and lead should never be used in the acute stage of gonorrhea, but should be reserved for the post-gonococcus period when the urine remains shreddy. 5. These astringents should not be used in sufficient strength to cause the patient to experience pain or irritation.

To summarize concerning chronic gonorrhea: 1. Endoscopic diagnosis and treatment is indispensable as a routine measure. 2. Silver nitrate or other caustic or irritating applications or instillations should be seldom used, and then only in the most skilled hands and with the greatest care; otherwise there are likely to occur structural changes in the urethra, predisposing to organic affections amenable only to surgical procedures.

The discussion of Dr. Loux's paper was opened by *Dr. H. M. Christian.* Dr. Christian complimented Dr. Loux upon the excellence of the paper read and stated that the methods mentioned were, in the main, those practiced by himself. He stated that potassium permanganate is of no value in gonorrhea other than as a simple cleansing agent. Dr. Christian agreed with Dr. Loux that argyrol was undoubtedly the best gonococcide known to-day.

After having used that silver salt continuously for more than two years, he prefers it because it never irritates, it is rapidly destructive to the gonococci, lessens the discharge and shortens the duration of the disease.

The speaker stated that it must be borne in mind that we have to deal not only with the gonococci, but with the destructive action of the micro-organism as well; in other words, destruction of the gonococcus does not by any means imply of necessity the cure of the disease, as there always remains a condition of catarrhal urethritis which requires a particular line of treatment. If a case of gonorrhea is seen in the early inflammatory stage, where ardor urinae and chordee are the most annoying subjective symptoms, Dr. Christian orders powders containing salol, sodium bromide, potassium bromide, each two and a half grains every two hours. At the same time a five per cent. solution of argyrol is ordered to be used by the patient as a hand injection three or four times daily, the solution being held in the urethra for ten minutes. If the patient can spare the time it is advisable to wash out the anterior urethra with several syringefuls of warm normal salt solution prior to using the argyrol injection; this line of treatment can be carried on through the second and third week. When the subjective symptoms subside it is sometimes of considerable advantage to supplement the local treatment with the use internally of copaiba and sandalwood oil. Ordinarily at the beginning of the third week, the patient enters upon the stage of decline, or as Professor Finger styles it, the "mucous terminal stage" of the disease. In a case going on to recovery the discharge is now scanty, then muco-purulent in character and containing few, if any, gonococci, and this is by far the most important stage in the treatment of the disease as regards the patient's future welfare; it is here that experience teaches that we need more than a mere gonococcidal agent. We need here in addition mild astringent lotions to help restore the integrity of the damaged mucous membrane. A good plan now is to use five per cent. solution of argyrol night and morning, employing through the day some such astringents as zinc, bismuth, hydrastic, lead, berberine, etc. At the beginning of the fifth week when nothing remains but the well-known "morning drop" and the urine is clear but contains shreds, it is well to use the argyrol solution at night, and to use once or twice through the day one of the well-known astringent mixtures.

If, in the second or third week the clinical symptoms and the two-glass test show involvement of the whole urethra, the treatment by hand injections is temporarily abandoned. Deep instillations of ten per cent. solutions of argyrol are then employed at short intervals until such time as the second urine becomes clear.

This in general is the line of treatment that the speaker had used at the University of Pennsylvania and his other clinics for the past two years, and is one that has given more satisfactory results than any hitherto employed.

*Dr. Orville Horwitz* condemned the irrigation treatment of gonorrhea and summarized his opinion as follows: The irrigation method of treatment will not abort acute specific urethritis; chronic urethritis and involvement of the deep sexual organs are common sequences; in many instances, in order to effect a cure in the terminal stage of the disease, the irrigation must be discontinued and other methods of treatment employed; irrigation should not be employed in the acute stage of specific urethritis; irrigation of the deep urethra by means of hydrostatic pressure is injurious in the majority of cases of acute gonorrhea, and is conducive to the development of complications; the best treatment we have to-day for gonorrhea is that by means of injection of argyrol solutions which are strongly antiseptic and non-irritating.

*Dr. R. O. Kevin* prefaced his remarks with the statement of the French surgeon Ricord, that "A clap begins and God alone knows when it will end." Fortunately, however, this statement is not as true as it was a few years ago. Dr. Kevin stated that with the use of 20 per cent. argyrol solution he had been able to cure 85 per cent. of his acute cases in from two to four weeks. He quoted Purdy of London, who stated that since the introduction of argyrol into the enormous clinic at the London Lock Hospital, 72 per cent. of the early cases had been cured within a month and there had been no relapses after six weeks. Dr. Kevin had been associated with Dr. Loux for several years at the clinic and could corroborate his conclusions. The speaker stated that he had seen so many acute cases cut short that he always practiced the abortive method when possible; this method is as follows: If the patient presents himself during the first forty-eight hours of an attack of gonorrhea, the anterior urethra is washed out with warm water or normal salt solution. Then two drachms of 20 per cent. argyrol solution is injected into the anterior urethra and held there for ten minutes; this injection is repeated by the patient every three hours, night and day, for three days. Even if this method does not abort the disease, it always effects a cure in a shortened period, and affords the patient entire freedom from the pain and irritation usually present in an early gonorrhea. If by this means the disease does not show signs of being aborted, Dr. Kevins practices the same methods mentioned by Dr. Loux.

*Dr. Orville Horwitz* read a paper entitled "The Radical Cure of Senile Hypertrophy of the Prostate; based upon a study of 145 operations performed by the author." Dr. Horwitz stated that the question under discussion has, with the possible exception of appendicitis, attracted more attention in the surgical world than any other subject. It is well-recognized that the danger to the patient with enlarged prostate begins as soon as it is necessary to resort to the daily use of the catheter, and when this period arrives a surgeon should be consulted to supervise the case and decide what operative measures are desirable or necessary. It was emphasized that no one operation was suitable to all cases, and that each patient is a law unto himself in the matter of choice of operation.

The two operations which have stood the test of experience are prostatotomy by means of the galvano-cautery (the so-called Bottini operation), and prostatectomy. The speaker stated that the Bottini operation is extremely valuable, safe, and always to be preferred to cutting operations in suitable cases. Out of ninety-eight cases operated upon by the author, by the Bottini method, three died—two of uremia and one of sepsis; all three were very old men. Twelve cases were lost sight of after leaving the hospital but were much improved when last examined. This leaves eighty-one cases concerning which there was obtained definite knowledge as to results. The ages of the patients varied between fifty-two and eighty-one years. The speaker stated that his statistics proved conclusively that the earlier the patient submitted to operation the better the results. Of the total eighty-one Bottini operations, all the patients were either entirely cured or very much benefited; four required second operation, and a considerable proportion were treated for several months subsequently for accompanying chronic cystitis.

Prostatectomy, the speaker stated, is regarded as a valuable operation, but authorities differ as to when and how it is to be performed. As many as twenty different operations have been suggested. Here, too, the individual case decides methods, choice of operation, etc. The prostatectomies performed by the author were as follows: three complete (suprapubic incision); six complete (combined suprapubic and perineal incisions); seven partial prostatectomies (suprapubic incision); thirty-four complete perineal prostatectomies.

Of the nine complete suprapubic operations, two died—one of suppression of urine, one of uremia. In all the cases convalescence was slow; in five cases the ultimate results were all that could be desired.

Of the thirty-four perineal prostatectomies, six died from uremia, sepsis or shock; six cases were lost sight of after leaving the hospital; sixteen were cured, four markedly benefited, one unimproved.

Dr. Horwitz summarized the results of observations in his 145 operations as follows:

1. A routine method is not applicable to the treatment of prostatic hypertrophy; every case is a law unto itself and the treatment will depend on the various conditions presented in each individual case.

2. The dangers attendant on the daily catheterism are greater than those of a radical operation performed at the onset of the symptoms caused by the obstruction.

3. The proper time to perform a radical operation is reached as soon as it becomes necessary for a patient to resort to daily catheterism.

4. The gratifying results obtained by a number of the operations in many cases demonstrates that the Bottini operation is one of great surgical value. It is applicable to a large percentage of cases, which if properly selected has proved to be the safest and best method of relieving an obstruction caused by prostatic hypertrophy. In those cases in which a stone in the bladder is associated with a prostatic enlargement, lithoplaxy may be performed in conjunction with a galvano-cautery prostatectomy.

5. A complete prostatectomy is justifiable if performed early before the individual is broken down in health and secondary complications have supervened. In early operations the results are most satisfactory, recovery rapid, the mortality varying between 5 per cent. and 7 per cent.

6. A complete prostatectomy in feeble elderly patients with long-standing obstruction and secondary complication, the prognosis is grave and the mortality ranges between 15 per cent. and 18 per cent. If the bladder in these cases happen to be hopelessly disabled, the results obtained by the operation are negative. Cases of this description are only suitable for suprapubic drainage.

7. In 90 per cent. of all cases the gland can be readily removed by means of a median perineal incision. The perineal operation recommended by Bryson is considered the operation of choice.

8. Complete suprapubic prostatectomy is shown to be more dangerous than the perineal operation for obvious reasons. A suprapubic prostatectomy is safer if combined with perineal drainage.

9. Partial suprapubic prostatectomy is indicated in such cases as where a valve-like lobe exists which interferes with urination, or where there is a partial hypertrophy of one of the lobes.

10. A perineal prostatectomy is best suited for those cases where the enlargement of the lateral lobes has a tendency to progress towards the rectum, to obstruct the urethra, or project backwards into the bladder.

11. A prostatectomy is always attended with more danger than the Bottini operation and the convalescence is more prolonged. In suitable cases the latter operation is therefore the one of choice.

*Dr. Edward Martin* discussed the preceding paper as follows: He agreed with Dr. Horwitz that, if operation has been advised and consented to, the circumstances of the individual case decided which of the several operations is to be performed. He believed that the Bottini operation has proved of great value and is preferable to cutting operations in suitable cases. He did not, however, advise operation in all cases of enlarged prostate. He recognized the inconveniences and dangers attendant upon the daily use of the catheter, but believed in the value of palliative measures in the majority of cases. He recommended care in the selection of catheters and chose one that enters the bladder with the least force and least pain to the patient. If a soft rubber catheter can not be introduced, a woven elbowed one is to be chosen. If obstruction or spasm necessitates habitual resort to a metal catheter, surgical intervention is required. When the patients use the instrument upon themselves, the hands should be washed thoroughly, dipped in bichloride solution, the meatus washed with the same solution, and be provided with an irrigating bag containing one pint of hot argyrol solution, 1 to 1,000. Infection of the bladder is commonly present and should be treated by means of bladder irrigations. For this purpose a fountain syringe, supplied with a catheter, should be suspended two feet above the level of the bladder. The anterior urethra is first thoroughly flushed, after which the catheter is pushed into the bladder and the urine withdrawn. The flushing of the bladder is continued until the return flow no longer contains pus or mucus. The temperature of the argyrol solution employed should be of the temperature of the body or a little above it. When practicable this antiseptic flushing should be done each time the catheter is passed. If this treatment is inefficacious continuous catheterization becomes necessary. For this purpose a large soft rubber catheter, or a self-retaining one, is selected and the antiseptic solution introduced; if the catheter is properly introduced, the entire amount of the solution will return. Twice a day the urethra and bladder are thoroughly flushed with the antiseptic solution, the catheter being withdrawn far enough to allow the injected fluid to escape from the meatus, and then being pushed back into its former position.

The success of this treatment depends upon securing free and continuous drainage, and this is incident to the permeability of the catheter and its retention in the proper position. When skillfully applied it is one of the safest and most successful means of treating cystitis, which so frequently complicates obstruction from prostatic enlargement.

